# The metabolism and significance of homocysteine in nutrition and health

**DOI:** 10.1186/s12986-017-0233-z

**Published:** 2017-12-22

**Authors:** Avinash Kumar, Henry A. Palfrey, Rashmi Pathak, Philip J. Kadowitz, Thomas W. Gettys, Subramanyam N. Murthy

**Affiliations:** 1 Environmental Toxicology Department, Southern University and A&M College, Baton Rouge, LA 70813 USA; 2 Department of Pharmacology, Tulane University School of Medicine, New Orleans, LA USA; 3 Laboratory of Nutrient Sensing and Adipocyte Signaling, Pennington Biomedical Research Center, Baton Rouge, LA USA

**Keywords:** Homocysteine, Methionine, Hyperhomocysteinemia, Inflammation, Dietary, Cardiovascular disease

## Abstract

An association between arteriosclerosis and homocysteine (Hcy) was first demonstrated in 1969. Hcy is a sulfur containing amino acid derived from the essential amino acid methionine (Met). Hyperhomocysteinemia (HHcy) was subsequently shown in several age-related pathologies such as osteoporosis, Alzheimer’s disease, Parkinson’s disease, stroke, and cardiovascular disease (CVD). Also, Hcy is associated with (but not limited to) cancer, aortic aneurysm, hypothyroidism and end renal stage disease to mention some. The circulating levels of Hcy can be increased by defects in enzymes of the metabolism of Met, deficiencies of vitamins B_6_, B_12_ and folate or by feeding Met enriched diets. Additionally, some of the pharmaceuticals currently in clinical practice such as lipid lowering, and anti-Parkinsonian drugs are known to elevate Hcy levels. Studies on supplementation with folate, vitamins B_6_ and B_12_ have shown reduction in Hcy levels but concomitant reduction in certain associated pathologies have not been definitive. The enormous importance of Hcy in health and disease is illustrated by its prevalence in the medical literature (e.g. > 22,000 publications). Although there are compelling data in favor of Hcy as a modifiable risk factor, the debate regarding the significance of Hcy mediated health effects is still ongoing. Despite associations between increased levels of Hcy with several pathologies being well documented, whether it is a causative factor, or an effect remains inconclusive. The present review though not exhaustive, is focused on several important aspects of Hcy metabolism and their relevance to health.

## Background

Homocysteine (Hcy) is a sulfur containing amino acid formed during the metabolism of methionine (Met) to cysteine (Cys). Hyperhomocysteinemia (HHcy), or increased circulating levels of Hcy, is generally recognized as an independent risk factor for coronary, cerebral, and peripheral atherosclerosis [1–3]. The levels of Hcy can be increased by defective metabolism of Met, resulting from either mutation in genes coding for the enzymes of Hcy metabolism [4, 5], or deficiencies of certain vitamin cofactors [6, 7]. In addition to genetic alterations, vitamin deficiencies, and several other environmental factors such as increased intake of Met, certain medications, disease state, pregnancy, and lactation are known to contribute to variations in Hcy levels [8–11]. Altered cellular export mechanisms have also been known to increase Hcy levels [12]. In general the dietary contribution of Hcy alone is insignificant given its low levels in most foods [13]; mainly derived from Met.

Serum or plasma Hcy is a well-known risk factor for cardiovascular disease (CVD) [14, 15] and this has been demonstrated among others in subjects with significant carotid stenosis particularly [16, 17]. It has been shown that HHcy following a Met load was significantly more in non-insulin dependent diabetes mellitus patients with vascular disease and also in patients with reduced cystathionine-β-synthase (CβS) activity [18, 19]. Studies have shown total-Hcy (tHcy) levels as a strong predictor of mortality in patients with angiographically confirmed coronary artery disease [20]. Association of elevated serum Hcy with sudden unexpected death, especially in patients with diabetes, has been reported [21]. In the absence of other known risk factors, HHcy was shown to stimulate the expression of monocyte chemoattractant protein-1 (MCP-1), vascular cell adhesion molecule-1 (VCAM-1), and E-selectin. This leads to increased monocyte adhesion to the arterial endothelium, and may significantly contribute to the development of atherosclerosis by facilitating monocyte/macrophage infiltration into the arterial wall [22].

Apart from defects in the enzymes of Hcy metabolism that are inherited, HHcy can also be induced experimentally by dietary manipulations. These include enrichment of diets with Met or depleting diets with vitamins folate or B_6_ or B_12_ [23]. Further, studies have shown that consumption of meat and dairy based products can also bring about an increase in circulating levels of Hcy [24, 25]. In a cross over study using an isocaloric diet but varying in protein concentrations, Verhoef and coworkers have shown increases in postprandial Hcy following the consumption of a high protein diet; 4–4.5 g protein bound methionine/d [26].

Hcy damages cells and tissues of arteries by eliciting the release of cytokines, cyclins, and other mediators of inflammation and cell division [27]. Additionally, Hcy causes oxidative stress by affecting cellular respiration, leading to oxidation of low density lipoproteins (LDL) and other constituents of plaques [28]. Stamler and colleagues reported that Hcy also antagonizes the vasodilatory properties of nitric oxide (NO) by forming *S*-nitrosohomocysteine, leading to endothelial dysfunction, the forerunner for atherogenesis [29].

Although Hcy has been associated with CVD and other age-related pathologies, whether it is a consequence or causative factor is subject of intense debate. The negative findings of interventional studies with vitamin B therapy have cast doubts on the role of Hcy in CVD pathogenesis resulting in Hcy controversy. To complicate this further, some interventional studies with folic acid and B vitamins have shown a reduction in Hcy levels but not the pathology, however these have met with criticisms in the design of the study that include duration and inadequate number of subjects [30]. It is argued that the Hcy lowering trials have been underpowered and also the duration of follow-up not being long enough; a consideration largely ignored. Interestingly, in a meta-analysis of the effect of B vitamins on stroke Wang and coworkers investigated the difference in outcome of follow-up for less than 36 months and more than 36 months. They demonstrated a statistically significant 29% reduction in the studies with at least 36 months of follow-up which could not be seen in the short-term studies [31]. Several studies on supplementation of folic acid to lower Hcy have not considered the possible adverse effects of unmetabolized folic acid (especially when given in large doses). Folic acid used for supplementation has to be reduced to tetrahydrofolate which requires 1-carbon substitution to initiate the methylation process. Further, it is argued that the beneficial effects of lowering of Hcy by B vitamins could depend upon the disease stage. Where, in the absence of atherosclerotic lesions lowering Hcy could be advantageous, in elderly patients with significant atherosclerotic lesions the B vitamins may well be neutralizing the benefits of lowering Hcy. The limitation in the duration of the intervention for reducing Hcy and related pathology especially those that develop and progress over several years like atherosclerosis may depend upon the severity of HHcy. Rather than abandoning the association of Hcy with CVD, revisiting the interventional approaches and interpretation of data may likely help in a better understanding of the relevance of Hcy to CVD.

## Historical

Hcy was first isolated from the urinary bladder stone(s) by Vincent du Vigneaud in 1933 [32]. Around the same time an 8-year-old boy of Irish-American ancestry admitted to Mass General Hospital was evaluated for 4 days for headache, vomiting and drowsiness with signs of poor mental development in addition to dislocation of lenses in both eyes. Severe deterioration in the boy’s condition with signs of stroke and weakness with abnormal reflexes on the left side were also reported [33]**.** Furthermore, although there were no signs of infection, there was a rise in blood pressure and temperature; the boy succumbed to the illness within few days. The cause of death was reported as arteriosclerosis of the carotid artery with cerebral infarct; published as case 19,471 in the New England Journal of Medicine in 1933 [34].

In 1965, a 9-year-old girl of Irish-American descent was evaluated in pediatric clinic of Mass General Hospital for slow mental development. This girl had a dislocation of her eye lens and exhibited several similarities with cases of homocysteinuria. This disease was just then discovered in Belfast, Northern Ireland while studying the chemical composition of the urine. The laboratory test on the girl’s blood sample confirmed HHcy [33]. The pediatricians became aware of her uncle who had died of a similar disease in childhood and due to the unusual nature of his case, it was published in a medical journal in the 1930s. Upon investigation, it was found that the reference made was to the report describing case 19,471 in the Nov 23, 1933 issue of NEJM.

In 1969, McCully a pathologist from Harvard ran into 2 children with homocysteinuria. The first one was a boy (only of 2 months of age) who had an advanced stage of arteriosclerosis as seen in older adults with advanced CVD. The laboratory analysis revealed extremely high levels of Hcy in blood and urine with no lipid deposits in their vascular plaques. The second instance was the autopsy tissue from an 8-year-old homocysteinuria child who had died of stroke; the tissue looked exactly like those of elderly men with arteriosclerosis. McCully therefore hypothesized that the vascular pathology observed in these patients could be the direct result of exposure to elevated levels of Hcy in the circulating blood. This was based on the observations of phenomenal increase in the levels of Hcy in addition to Met, cystathionine, homocystine, mixed disulfide of Hcy and Cys. Based on these observations, he suggested for the first time that elevated Hcy was a likely cause of premature vascular disease [1]. The medical community however did not accept his hypothesis for a long time and certainly not until similar observations were made and confirmed by others several years later [35].

## Biosynthesis and metabolism of Hcy

Met is the sole source of Hcy which can reform Met (remethylation pathway) or be metabolized to Cys (transsulfuration pathway) or can cyclize to form homocysteine thiolactone (HTL). Also, Hcy can be considered as an intermediate in the SAM cycle. The first step in the transfer of methyl group is the reaction of Met with ATP resulting in the formation of S-Adenosyl-L-methionine (AdoMet or SAM); a universal methyl donor. After donating the methyl group to acceptor molecules (DNA, RNA, amino acids, proteins, phospholipids etc.), the resulting demethylated compound formed is S-Adenosyl homocysteine (SAH). This SAH then undergoes deadenosylation (S-adenosyl homocysteine hydrolase mediated) resulting in the formation of Hcy. The Hcy so formed can be remethylated back to Met (methionine synthase; vitamin B_12_ dependent) utilizing the methyl group from 5-*N*-methyl tetrahydrofolate. This route of Hcy metabolism is termed the remethylation pathway. Alternatively, Hcy can combine with serine to form cystathionine. This reaction is catalyzed by CβS which is a vitamin B_6_ dependent process. This route of the metabolism of Hcy is termed as the transsulfuration pathway.

In addition to remethylation and transsulfuration pathways, Hcy can undergo cyclization to form HTL, and this thioester is considered to be the toxic intermediate of Hcy. The formation of HTL is documented to be due to an error-editing reaction in protein biosynthesis. Owing to the structural similarity between Met and Hcy, methionyl-tRNA synthetase takes up Hcy instead of Met. However, this error is immediately edited where the AMP is lost from the activated Hcy or adenylated Hcy (not S-adenosyl-Hcy) resulting in the cyclization process [36]. Among others, accumulated levels of Hcy/defects in the metabolism of Hcy, have been considered to potentiate the formation of HTL as documented [37].

**Fig. 1:**
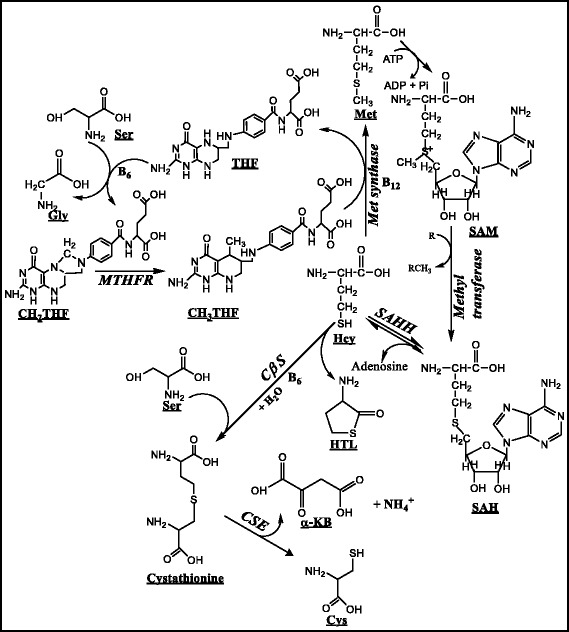
Metabolism of Homocysteine. The metabolism of methionine is depicted only till the formation of cysteine. Homocysteine can be either remethylated to methionine (Remethylation pathway) or can be routed for the formation of cysteine (Transsulfuration pathway). Cys- Cysteine, Gly- Glycine, Hcy- Homocysteine, HTL- Homocysteine thiolactone, SAH- S-adenosyl-L-homocysteine, SAM- S-adenosyl-L-methionine, THF- Tetrahydrofolate, CH_2_THF- Methylene tetrahydrofolate, CH_3_THF- Methyl tetrahydrofolate, Met- Methionine, Ser- Serine, α-KB- α-ketobutyrate, MTHFR- Methylene tetrahydrofolate reductase, CβS- Cystathionine β synthase, CSE- Cystathionine γ lyase, SAHH- S-adenosyl homocysteine hydrolase

Although 5-*N*-methyl tetrahydrofolate is the major source for methyl group in the remethylation of Hcy to Met, betaine and choline also can serve as methyl donor molecules. The betaine pathway however is mainly restricted to the liver and is mediated by betaine Hcy methyl transferase. The metabolism of Met to Hcy, Cys and HTL is depicted in Fig. 1.

## Biosynthesis and metabolism of hydrogen sulfide

**Fig. 2:**
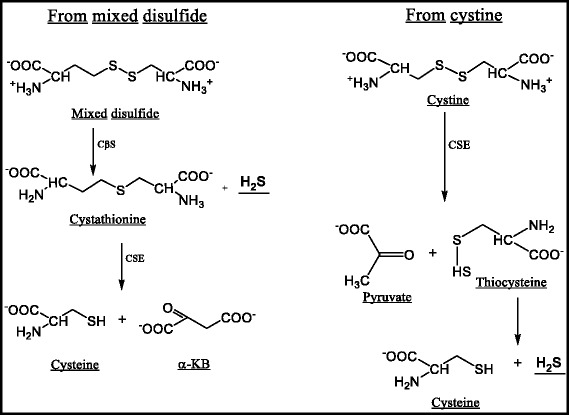
Formation of Hydrogen Sulfide in vivo*.* The in vivo formation of Hydrogen sulfide is presented. This can be formed from the disulfide of cysteine-Cystine (Cys-Cys) or the mixed disulfide of cysteine and homocysteine (Cys-Hcy). α-KB- α-ketobutyrate, CβS- Cystathionine β synthase, CSE- Cystathionine γ lyase, H_2_S- Hydrogen sulfide

Hydrogen sulfide (H_2_S) is formed by the enzymatic action of CβS during the metabolism of the mixed disulfide (Hcy-Cys) to cystathionine (Fig. 2). Alternatively, H_2_S can also be formed from cystine (disulfide of cysteine) via enzymatic action of cystathionine-γ-lyase (CSE), resulting in the production of thiocysteine and pyruvate with the liberation of NH_3_. The thiocysteine is subsequently cleaved to H_2_S and Cys (Fig. 2). Similar to NO and carbon monoxide (CO), H_2_S is a lipid-soluble gaseous messenger molecule [38]. These 3 gas molecules make up the family of labile biological mediators termed gasotransmitters. Till recently these gases were considered to be highly toxic apart from the concerns as environmental hazards. The discoveries that these molecules are enzymatically regulated and endogenously produced under normal physiological conditions in mammals have prompted re-evaluation of the biological role of H_2_S, NO and CO. These gasotransmitter molecules have also been extensively studied that indicate that the enzymes that generate them share similar features and overlap in a variety of biological functions [39]. Whereas deficiencies in the enzymes (through genetic manipulation or use of inhibitors) exacerbate ischemia-reperfusion (I/R) injury, overexpression of these enzymes or treatment with pharmacological donors or inhaled gas therapy have been shown to have cytoprotective effects.

Using a rat model of I/R, Xu and coworkers [40] have demonstrated a marked reduction in the levels of H_2_S in the kidney and that impairment in the activity of CβS was responsible for reduction in renal H_2_S synthesis. These workers also demonstrated that partial restoration of CβS activity by an NO scavenger increased renal H_2_S levels and also improved renal function. There is supportive evidence that H_2_S production from vascular tissues is enhanced by NO [39, 40]. Upon administration of sodium hydrosulfide (NaHS) a H_2_S donor, it was shown that there was a reduction in I/R induced kidney injury. Further, it helped in improving the organ function demonstrating that restoration of CβS -mediated H_2_S synthesis could exert a renal protective effect against I/R injury [40].

Functionally, H_2_S has also been shown to induce hippocampal long-term potentiation, brain development, and facilitate blood pressure regulation. By acting specifically on ATP-sensitive potassium (K_ATP_) channels, H_2_S can hyperpolarize cell membranes, relax smooth muscle cells, or decrease neuronal excitability [41]. Endothelial cells produce and release H_2_S in a Ca^2+^-dependent manner following neurohumoral stimulation, and evokes hyperpolarization and relaxation of vascular smooth muscle cells by activating K_ATP_ channels [42–44]. The cardiovascular effects of H_2_S (antioxidant properties, vasodilation, and a decrease in blood pressure) have been linked to the activation of CβS by calcium-calmodulin complex. Genetic deletion of *Cbs* or *Cse* gene in mice markedly reduces H_2_S levels in the serum, heart, aorta, and other tissues, resulting in pronounced hypertension and diminished endothelium-dependent vasorelaxation. Such observations support the concept that H_2_S is a physiologic vasodilator and regulator of blood pressure [45].

In attempts to explore mechanisms by which H_2_S elicits its vasodilatory properties, the cardiovascular responses of H_2_S donors, sodium sulfide (Na_2_S) and NaHS, have been examined in the anaesthetized rat. These agents while eliciting a hypotensive effect also brought about a decrease in heart rate, systemic arterial pressure, and cardiac output. The attenuation of these effects were not caused by haxamethonium (nicotinic receptor antagonist), glybenclamide (K_ATP_ channel antagonist), sodium meclofenamate (cyclooxygenase inhibitor), miconazole (P_450_ epoxygenase inhibitor), N^ω^-nitro-L-arginine methyl ester (L-NAME) hydrochloride (nitric oxide synthase inhibitor), tetraethylammonium (Ca^2+^ activated, K^+^ channel inhibitor), and 1H-[1,2, 4]oxadiazolo[4,3,-a]quinoxalin-1-one (soluble guanylyl cyclase inhibitor). Additionally, the decreases in heart rate were not blocked by atropine, an anticholinergic agent, which indicates that the lowered heart rate was independent of parasympathetic activation [46].

## Significance of HTL

The metabolic conversion of Hcy to HTL, the reactive anhydride of Hcy was proposed by Jakubowski in 1997 [47]. The thioester HTL is formed as a byproduct of protein biosynthesis. As mentioned earlier, owing to structural similarities between Hcy and Met, the methionyl tRNA synthetase incorporates Hcy instead of Met during protein biosynthesis. However, in an error editing reaction it is edited resulting in the formation of HTL. There are emerging evidences documenting an important role for HTL in atherothrombosis. HTL forms isopeptide bonds with protein lysine (Lys) residues. Unlike a peptide bond (a bond between α amino and α carboxyl group of amino acids), in an isopeptide bond the linkage is between a non-α-amino and a α carboxyl group or a non-α-carboxyl and an α amino group of amino acids. The isopeptide bond leads to impaired or altered protein function and has pathophysiological effects including activation of an autoimmune and enhanced thrombosis response. Reaction of HTL with serum proteins leads to production of new protein antigens and autoimmune antibodies enabling inflammatory processes. Autoantibodies against Nε-Hcy-Lys-proteins occur in human plasma and positively correlate with plasma tHcy and has been associated with stroke in humans [48]. HTL mediated protein modification brings about changes in protein sequence which likely disrupts protein folding, and produces altered proteins with newly acquired interactions, thereby impacting the cellular physiology to a great extent. Furthermore, HTL interacts with LDL, causing aggregation, increase in density, and uptake by vascular macrophages to form foam cells [49]. Mammalian organisms, including humans, have evolved the ability to eliminate HTL and one such mechanism involves paraoxonase 1 (PON1), a high-density lipid (HDL) associated protein. There are three human PON proteins (PON1, PON2, and PON3), sharing about 65% gene similarity. The enzyme PON1 is a Ca^2+^-dependent esterase, and has the ability to hydrolyze HTL. While PON2 and PON3 are restricted to intracellular compartments, PON1 protein is predominantly secreted and found in circulation [50]. Recently a human hepatic enzyme was purified and identified as biphenyl hydrolase-like protein (BPHL). Both native and recombinant purified BPHLs could hydrolyze HTL with much higher catalytic efficiencies than that of purified plasma PON1 [51].

Perna et al. have documented protein-N-homocysteinylated derivatives with either free N-terminus or the ε-amino group of Lys residues [52]. Such Nε-Hcy-Lys-proteins lose their function and become susceptible to additional damage by oxidation. Human plasma levels of N-linked protein Hcy have been directly related to plasma tHcy levels [48]. Several proteins including hemoglobin, serum albumin, γ-globulins, LDL, HDL, transferrin, antitrypsin, and fibrinogen have been found to contain small amounts of N-linked Hcy.

Developmental abnormalities in chick embryos with optic lens dislocation have been reported when treated with HTL [53]. Additionally, in rats and baboons atherosclerotic changes have been reported upon feeding a diet supplemented with HTL implying pathophysiological changes similar to those observed in human genetic HHcy [54, 55].

## Estimation of Hcy

The normal levels of Hcy range between 5 and 15 μmol/L. Based on the levels of Hcy, HHcy has been classified as mild/moderate (15–30 μmol/L), intermediate (30–100 μmol/L) and severe (>100 μmol/L) [56–59]. Typically, Hcy in circulation is found either in free form (about 1%) or disulfide (Hcy-Hcy; homocystine) or mixed disulfide (Hcy-Cys) or in protein bound form. Therefore, in order to get a true estimate of Hcy, the free, disulfides and protein bound form of Hcy have to be accounted for. The expression total Hcy or tHcy is used to reflect the sum total of Hcy that comprises free Hcy, Hcy obtained from reduction of disulfides and Hcy liberated upon hydrolysis of proteins (protein bound).

Highly sensitive and reproducible methods for the analysis of Hcy are routinely used, and these include high performance liquid chromatography (HPLC), liquid chromatography-mass spectrometry (LCMS) and immunoassays to mention some. Each of these methods has its own advantages, however, the estimation of Hcy by HPLC which is most commonly used [60] is described in detail to illustrate approaches of accounting for all forms of Hcy to reflect tHcy in the sample.

Typically, for estimation of Hcy by HPLC in plasma/serum or tissue homogenates, the samples are subjected to a reduction reaction using reagents like tri-n-butylphosphine that renders the SH/thiol group free. This is followed by labeling with a thiol specific fluorescent reagent 4- (aminosulfonyl)-7-fluoro-2,1,3–benzoxadiazole (ABDF). Immediately, the protein in the sample is precipitated and the supernatant and precipitate are separated. The supernatant containing free and reduced forms of Hcy labeled with ABDF can be used for analysis by HPLC. The pellet of precipitated proteins is subjected to hydrolysis by conventional methods to liberate protein bound and ABDF labeled Hcy for analysis by HPLC. The Hcy values from supernatant and protein hydrolyzed fractions are added up to obtain tHcy. Most HPLC fluorometric methods use cysteamine as internal standard and all thiol containing compounds like Cys, Hcy and the tripeptide glutathione and the metabolite of glutathione, cysteinyl glycine can be separated and quantitated. This method helps in interpreting the exact value of Hcy in serum and tissue samples.

## Pathologies associated with Hcy

Numerous studies have demonstrated an association of HHcy with vascular disease [1–3, 15, 35, 61–63], and several age related pathologies like Alzheimer’s disease [64–66], stroke [67, 68], Parkinson’s disease (PD) [66] have also been linked to high levels of Hcy. Additionally there are studies showing an association between HHcy and osteoporosis [69], end stage renal disease (ESRD) [70], insulin resistance (IR) [71, 72], aneurysm [73–75], hypothyroidism [76, 77] cancer [78, 79] and gastrointestinal disorders [80] to mention some.

### Vascular disease and mechanism of endothelial dysfunction

Elevated levels of Hcy are involved in the development and progression of vascular disease. It is known that HHcy causes endothelial dysfunction and has been attributed to be due to impaired bioavailability of NO [81]. One likely mechanism for reduced bioavailability of NO is mediated by asymmetric dimethylarginine (ADMA). This endogenous inhibitor of endothelial nitric oxide synthase (eNOS) competes with the natural substrate, L-arginine thus limiting the formation of NO. Elevated plasma levels of ADMA have been associated with HHcy and endothelial dysfunction in both animals and humans [82]. Apart from inhibiting the production of NO, ADMA may also promote the “uncoupling” of eNOS, thereby increasing the production of superoxide and other reactive oxygen species which in turn may further decrease NO bioavailability.

Protein arginine N-methyltransferases (PRMTs) 1 and 2 are involved in the methylation of protein arginine residues. Typically, there can be monomethylated arginine or symmetrically methylated dimethyl arginine (mediated by PRMT 2) or asymmetrically methylated dimethyl arginine (PRMT 1 catalyzed). PRMTs utilize SAM as a methyl donor and generate SAH (and ultimately Hcy) as a byproduct. The major pathway for metabolism of ADMA is the formation of citrulline and methylamine mediated by dimethylarginine dimethylaminohydrolase (DDAH) [83]. A small amount of ADMA is also metabolized to α-keto acids or excreted by the kidney. It has been surmised that Hcy enhances the activity of PRMT 1 and also increases the proteolysis of proteins with methylated arginine residues. Similarly, Hcy can influence the activity of DDAH which would prevent the metabolism of ADMA and thus bring down the levels of NO. Also, Hcy may elevate ADMA levels by inducing ER stress and cell death, leading to increased proteolysis of proteins that contain methylarginine residues. The likelihood of HHcy enhancing PRMT activity is controversial since it results in high levels of SAH; a potent inhibitor of SAM-dependent methyl transfer reactions. It remains possible, however, that the inhibitory effect of high levels of SAH in HHcy might compensate for the increased expression of PRMT 1 [82, 84].

### Alzheimer’s disease, stroke and Parkinson’s disease

Elevated plasma tHcy has been linked to cognitive decline, white matter damage, brain atrophy, neurofibrillary tangles, and dementia. In a mouse model, Hcy has been shown to exacerbate β-amyloid plaque formation, the process related to the onset of Alzheimer’s disease [85]. Changes to blood brain barrier integrity and function are also suggested to result from elevated blood levels of Hcy [86]. A frequently encountered problem in ageing populations with hypertension is an apparent association between HHcy and stroke. In individuals who have stroke, blood supply to the brain is interrupted resulting in neuronal cell death and loss of function in specific brain regions, and has been attributed to be due to the lack of oxygen and nutrients [87]. An increase of tHcy levels in both plasma and cerebrospinal fluid has been attributed to result in cognitive decline and depression in patients of PD. This is a progressive neurological disorder that greatly affects movement, and over 3–5% of the US population of 65 years and older suffer from this pathology. Persons clinically presenting this disease would normally show signs such as resting tremors, muscular rigidity, and postural instability. In PD, Hcy mediated oxidative stress/damage in dopaminergic neurons of substantia Nigra is commonly seen; hallmark of the disease. The oxidative stress in dopaminergic neurons caused by Hcy has been recently reported [88].

### Osteoporosis

It is documented that Hcy affects bone mineral density and remodeling in a way that bone matrix is altered and osteoclast activity is stimulated [89, 90]. This could result in bones becoming less rigid, increasing the chances of fracture. In a study by Yilmaz and Eren, Hcy was suggested to alter bone mineral density by adversely affecting antioxidant capacity of osteoclasts in postmenopausal women [90]. Blood flow to bone is exceptionally important since it carries all the minerals and nutrients for ossification. Therefore, in order for bone to repair/recover from injury adequate blood supply is crucial, as impeding blood flow can greatly enhance osteoporotic conditions [91]. Additionally, a study by Tyagi et al. found decreased bone blood flow in *Cbs* knockout mice with or without folic acid supplementation [92].

### End stage renal disease (ESRD)

There is a strong association between ESRD and HHcy. The deterioration in renal function with uremia has been observed, and was associated with high blood levels of Hcy. The common causes known to contribute to this pathology are high blood pressure and diabetes. Chronic renal failure and uremia represent states wherein high blood levels of Hcy are seen [70], and with decline in function these patients become more and more hyperhomocysteinemic [93]. Also, an association between dialysis and an increase in plasma level of Hcy is documented [94]. Dialysis has been shown to lower Hcy levels, albeit transitorily.

### Insulin resistance (IR)/ diabetes

According to a population based study, raised maternal plasma tHcy concentrations predict small size at birth, which has been considered a risk factor for type 2 diabetes mellitus (T2DM) [95]. Vitamin B_12_ deficiency is a commonly encountered problem and contributes to HHcy. An association between maternal vitamin B_12_, folate and tHcy status during pregnancy was studied and correlated with offspring adiposity and IR at 6 years of age. Children born to mothers with low vitamin B_12_ but high folate concentrations were the most insulin resistant. It has been hypothesized that in utero vitamin B_12_ and folate deficiency could predict about propensity for greater adiposity and insulin resistance in the offspring.

An association between elevated levels of Hcy and insulin resistance/diabetes is well documented. T2DM is a chronic inflammatory disease characterized by increased levels of blood glucose, and insulin. IR which generally precedes diabetes is a pathological condition in which the capacity of cells to respond to normal levels of insulin declines thus forcing the body to produce more and more insulin to be able to prevent hyperglycemia. The insulin signaling is an important process which when impaired causes IR. Among others, Hcy is known to disrupt insulin signaling by interfering with the phosphorylation of insulin receptors and thereby impacting the downstream signaling cascade. This ultimately results in lowered GLUT4 translocation or recruitment on to the plasma membrane and therefore reduced glucose uptake. Further, the impairment in signaling was followed by increased production of resistin; a peptide hormone of adipose tissue. Resistin has been associated with obesity which can be further linked to diabetes [96]. A direct association between HHcy and IR is well demonstrated by Golbahar and colleagues using Sprague Dawley (SD) rats [71]. Another important factor that contributes to IR is endoplasmic reticulum (ER) stress. A clear association between HHcy and ER stress, where an elevation in variant ER stress markers in adipose tissue of homocysteinemic mice has been demonstrated [97].

### Other pathologies

HHcy, an independent risk factor for vascular disease has been shown to occur in other related pathologies as well. Abdominal aortic aneurysm (AAA) is a condition where abdominal aorta fails to dilate normally and studies report an association between elevated plasma Hcy levels and AAA [74, 75]. Similarly, increased levels of Hcy has also been seen in hypothyroidism and this has been associated with increased risk of CVD [76, 77]. In cancer patients, elevated Hcy levels may be caused due to rapidly dividing tumor cells and thus high plasma Hcy, a risk factor for cancer is a potential tumor marker [78]. More recently, HHcy has been shown to promote hepatocellular carcinoma via an epigenetic mechanism involving cytochrome P450 (Cyp450) metabolism [79]. It has also been implicated to adversely affect intestinal vasculature resulting in a condition such as inflammatory bowel disease characterized by chronic inflammation of the gastrointestinal tract [80].

There are ongoing efforts to understand if HHcy observed is due to the pathologies or if HHcy itself is causing the pathology. Several interventional approaches to alleviate HHcy, and its related pathologies have yielded conflicting results. According to Hope (Heart Outcomes Prevention Evaluation) 2 study, daily administration of the combination of folate, vitamins B_6_ and B_12_ significantly reduced Hcy levels but not the incidence of deaths from CVD, myocardial infarction (MI), and stroke [98]. These findings were in agreement with the NORVIT (Norwegian Vitamin) trial which concluded that folic acid with or without high doses of vitamin B_6_ did not reduce CVD or death after an acute MI, in spite of adequate Hcy lowering [99]. Similarly, there was no treatment benefit in VISP (Vitamin Intervention for Stroke Prevention) and VITATOPS (VITAmins TO Prevent Stroke) studies [100, 101]. On the other hand, there are also interventional trials which support the notion that targeting to lower Hcy will lower related pathologies. In a randomized controlled trial by Smith and coworkers, it was shown that brain atrophy in cognitively impaired elderly declined with B vitamin supplementation and the rate of which was dependent on pre-existing plasma ω-3 fatty acids [102]. Another large randomized CSPPT (China Stroke Primary Prevention Trial) study conducted in Chinese adults with hypertension without a history of stroke or MI, showed that oral folic acid supplementation with enalapril significantly reduced the relative risk of first stroke by 21% [103]. Similarly, a duration-based reduction (29%) in stroke was demonstrated after 36 months of follow-up against less than 36 months in a meta-analysis of the effect of B vitamins on stroke [31].

Apart from several age-related pathologies, HHcy has also been shown to play a role in depression, migraine and retinal vein occlusion. High levels of Hcy have been considered to cause cerebral vascular disease and neurotransmitter deficiency resulting in depression of mood. High Hcy may result in diminished remethylation to methionine; critical for the synthesis of epinephrine [104, 105]. Interestingly, in migraine, the cerebrospinal fluid levels of Hcy have been shown to raise remarkably [106]. In patients with retinal vein occlusion increases in Hcy levels have been reported which is in line with a report of a meta-analysis of an association between methylenetetrahydrofolate reductase C677T polymorphism and the risk of retinal vein occlusion [107, 108]. Increases in Hcy levels could result from a) lowered intake or deficiencies of vitamins B6, B12 and Folic acid or b) due to mutations in the enzymes of Met/Hcy metabolism or c) high intake of Met. Although high levels of Hcy have been associated with several pathologies, it could serve more as a marker than a true risk factor. The high levels of Hcy being the cause or effect of several pathologies is an ongoing debate.

## Therapeutic options

For the therapeutic management of HHcy, different approaches have been considered. Individual supplementation with folic acid, vitamin B_12_ and their combination with vitamin B_6_ to lower Hcy levels have been well studied. There have been reports of both beneficial and neutral effects with this therapeutic regimen [98, 109]. While both folic acid and vitamin B_12_ are required for normal Met synthase activity, vitamin B_6_ serves as a key cofactor necessary for the activity of CβS. Supplementation of folic acid, vitamin B_12_ and vitamin B_6_ were effective in lowering elevated plasma Hcy levels. Further, an improvement in endothelium-dependent vasodilation and reduction of exercise-induced myocardial ischaemia was demonstrated in patients who had HHcy and coronary heart disease [110]. Vitamin supplementation may be a cost-effective therapy devoid of adverse effects.

Along with methyl tetrahydrofolate, choline, an essential nutrient, also serves as a source of methyl groups. Upon oxidation choline forms betaine, which can then remethylate Hcy by donating one of its methyl groups resulting in the formation of Met and dimethyl glycine. This reaction is catalyzed by betaine-Hcy methyltransferase (BHMT) and this pathway is mainly restricted to liver and kidney. There are several studies that show lowering of tHcy by dietary supplementation of choline and betaine. This effect has been also shown to be independent of intake of folate and B vitamins and hence could be a plausible treatment option for lowering Hcy levels [111–114].

Nebivolol is a selective β-1 receptor blocker with NO enhancing properties, and is currently used in the clinical management of hypertension. A recent study demonstrates the beneficial effect of nebivolol in improving HHcy induced oxidative stress of different rat tissues. Further, it was shown to significantly lower Hcy levels and thereby providing an additional therapeutic option for lowering tHcy [115].

Glutathione is a naturally occurring tripeptide (γ-glutamyl-cysteinyl-glycine) with powerful antioxidant effects, and has been shown to play a major role in combating the oxidative stress. In view of the oxidative damage caused by Hcy, antioxidant therapies have also been considered. N-acetyl cysteine (NAC), has been used to replenish the depleting glutathione levels and thus has been used as a therapeutic option for combating HHcy [116, 117]. Among others, NAC has been widely used for several years in the treatment of chronic obstructive pulmonary disease. In patients undergoing cardiac angiography NAC has been shown to improve renal function, and together with folate, NAC is reported to lower plasma Hcy levels and improve endothelial function.

It is relevant to mention the influence of some of the common drugs which are in clinical practice that significantly affect the metabolism of Met resulting in increase of circulating levels of Hcy. Lipid lowering drugs like niacin and particularly fenofibrate, a peroxisome proliferator-activated receptor (PPAR) α agonist cause up to 50% increase in Hcy levels [118–120]. Rosiglitazone used in the management of hyperglycemia is one of the PPARγ agonist that has been shown to have pleiotropic effects. Interestingly, rosiglitazone has been shown to reduce tHcy levels in SD rats on a Met enriched diet [121]. Apart from some of the lipid lowering drugs, long term use of commonly used anti-epileptic drugs like phenytoin, carbamazepine and valproic acid have been shown to cause HHcy [122]. Additionally, L-DOPA treated patients of PD develop HHcy which has been implicated with cognitive dysfunction and dementia in these patients [123].

Improving therapeutic options for reducing pathologies associated with increased tHcy levels is an ongoing effort. Screening of patients on drugs for the management of depression/behavior and also on drugs that affect the metabolism of either B vitamins or essential amino acids could serve as a preventive measure in reducing the likelihood of HHcy. The consideration to screen Hcy levels for clinical management of certain diseases may therefore be important.

## Conclusions

In conclusion, whether HHcy is a cause for several pathologies or an effect needs additional understanding and more mechanistic studies. An alteration in the metabolism of Met which results in HHcy is associated with risk for vascular disease as well as several other age-related pathologies. Although successful restoration of Hcy to normal levels by intervention with B-vitamin supplementation has been realized, concomitant reductions in CVD risk were not seen in several investigations. Studies have shown that Hcy-induced impairment in the transportation of L-arginine (precursor of NO) into endothelial cells and up-regulation in the generation of reactive oxygen species by NADPH oxidase results in uncoupling e-NOS; culminates in reduced NO bioavailability and inflammation. Importantly, HHcy mediated induction of PRMT 1 and thence increase in the levels of ADMA together with the inhibition of DDAH by Hcy could contribute to the reduced bioavailability of NO. Further, the generation of HTL that attacks Lys-rich proteins and potentially trigger ER-stress-related endothelial apoptotic response contributing to endothelial dysfunction is well documented. Such modified proteins are known to initiate an autoimmune response and has been considered to be responsible for the initiation and progression of several disease processes. The increased incidence of HHcy in the population and its association with various diseases merits further understanding of the role of dietary approaches and alternative therapeutic modalities for the management of HHcy. It would be worthwhile to investigate if several pathologies attributed to HHcy are indeed due to Hcy alone or is due to the imbalance of other amino acids (during HHcy) that contribute and exacerbate responses seen in HHcy. Unraveling the mechanistic aspects of Hcy imbalance, as well as the need for the management of Hcy oscillations especially in disease states continues to be an active area of research that warrant a better insight. Despite over 22,000 research publications till now, the ambiguity about the importance of Hcy as a cause or effect in several diseases remains to be well established. The duration of intervention, the disease stage and the number of subjects to arrive at statistically significant and reproducible results for reducing Hcy and related pathologies may need more attention to clarify the association between Hcy and CVD.

## Abbreviations

AAA: Abdominal aortic aneurysm
ABDF: Aminosulfonyl)-7-fluoro-2,1,3–benzoxadiazole
ADMA: Asymmetric dimethylarginine
ATP: Adenosine tri phosphate
BPHL: Biphenyl hydrolase-like protein
CO: Carbon monoxide
CSE: Cystathionine-γ-lyase
CVD: Cardiovascular disease
Cys: Cysteine
CβS: Cystathionine beta synthase
e-NOS: Endothelial nitric oxide synthase
ESRD: End stage renal disease
H_2_S: Hydrogen sulfide
Hcy: Homocysteine
HHcy: Hyperhomocysteinemia
HPLC: High performance liquid chromatography
HTL: Homocysteine thiolactone
IR: Insulin resistance
LDL: Low density lipoprotein
Lys: Lysine
Met: Methionine
MI: Myocardial infarction
NAC: N-acetyl cysteine
NO: Nitric oxide
PD: Parkinson’s disease
PON: Paraoxonase
PRMT: Protein arginine N-methyltransferase
SAH: S-adenosyl-L-homocysteine
SAM: S-adenosyl-L-methionine
tHcy: Total homocysteine
